# Nanoemulsified Corn Oil in Lactating Barki Nutrition: Effect on Intake, Nutrient Digestibility, Rumen Fermentation Characteristics, and Microbial Population

**DOI:** 10.3390/ani15101424

**Published:** 2025-05-14

**Authors:** Min Gao, Rong-Qing Li, Mostafa S. A. Khattab, Ahmed M. Abd El Tawab, Yong-Bin Liu, Mohamed El-Sherbiny

**Affiliations:** 1 State Key Laboratory of Reproductive Regulation and Breeding of Grassland Livestock, Inner Mongolia University, Hohhot 010021, China; min.gao@imu.edu.cn (M.G.); rong-qing.li@mail.imu.edu.cn (R.-Q.L.); 2 National Sheep Genetic Evaluation Center, Inner Mongolia University, Hohhot 010070, China; 3 Department of Dairy Science, National Research Centre, 33 Bohouth St., Dokki, Giza 12622, Egypt; msakhattab@gmail.com (M.S.A.K.); amaeid2010@gmail.com (A.M.A.E.T.); 4 Department of Animal Genetics, Breeding, and Reproduction, College of Animal Science, Inner Mongolia Agricultural University, Hohhot 010018, China; 5 Institute of Animal Nutrition, Nutrition Diseases and Dietetics, Faculty of Veterinary Medicine, University of Leipzig, An den Tierkliniken 9, 04103 Leipzig, Germany

**Keywords:** oil, nanoemulsion, volatile fatty acid, unsaturated fatty acid, biohydrogenation, rumen bacteria

## Abstract

Nanoemulsified corn oil was tested on twenty-one multiparous lactating Barki ewes (mean ± SD: 3 ± 0.4 parity, 44.3 ± 1.9 kg body weight, 30 ± 2.7 months of age, and 402 ± 23 g/d of prior milk production) randomly allocated to the following treatments (*n* = 7 ewes/group): Control—a basal diet consisting of 50% concentrate mixtures and 50% berseem clover; CO—the Control diet + 3% of corn oil; NCO—the Control diet + 3% of nanoemulsified corn oil. A completely randomized design of 25 days of adaptation and 5 days of sampling was employed with seven ewes per treatment. Despite feeding oil according to the recommended values, CO decreased the dry matter intake by 8.3% and 6.7% compared to the Control and NCO, respectively. The negative impact of CO extended to reducing the concentrations of ammonia and total volatile fatty acids in the rumen. On the other hand, NCO had less effect on the biohydrogenation intermediates profile compared to CO; noticeably, higher proportions of unsaturated fatty acid (UFA) were associated with NCO; these results were also supported by an increase in the rumen microbial population with NCO compared to CO, especially the biohydrogenation bacteria, which showed higher abundance with NCO despite the low presence of biohydrogenation intermediates. In conclusion, the NCO demonstrated the ability to decrease the transformation of unsaturated fatty acids into saturated fatty acids in the biohydrogenation environment. This effect was not associated with decreased dry matter intake, changes in nutrient digestibility, or alterations in fermentation patterns.

## Simple Summary

Incorporating poly-unsaturated fatty acids into diets is essential for enhancing the functional attributes of ruminant products (meat or milk). Oils have been a preferred option for that aim. However, innovative methods are needed to provide unsaturated fatty acid-rich oils to the rumen in a manner that is less harmful to rumen bacteria compared to the traditional raw supplementation. The following experiment intended to compare raw corn oil (CO) with nanoemulsified corn oil (NCO) administered at 3% of the dry matter fed to dairy sheep. Compared to Control and NCO, the CO reduced dry matter intake, ammonia, and total volatile fatty acid concentrations in the rumen. Nonetheless, NCO exerted a lesser influence on the biohydrogenation of fatty acid intermediates compared to CO. Increased accumulation of unsaturated fatty acids (UFA) occurred in the rumen when NCO was administered. The population of rumen microorganisms in the rumen remained mostly unaltered by NCO, particularly the biohydrogenation bacteria. In conclusion, feeding NCO circumvented the biohydrogenation process that typically converts unsaturated fatty acids into their saturated form. This enhancement was not accompanied by alterations in dry matter intake, nutrient digestion, or ruminal fermentation pattern.

## 1. Introduction

In humans, fat from dairy products accounts for roughly 25–35% of the total daily intake of saturated fatty acids (SFAs) and up to 15–25% of the total daily fat consumption [1]. Ruminant milk fat comprises around 70% saturated fatty acids (SFAs), 8% trans fatty acids, and under 5% long-chain unsaturated fatty acids (LCUFAs). Elevated saturated fatty acid consumption correlates with a heightened risk of cardiovascular disease, obesity, and atherosclerosis [2]. Conversely, LCUFAs—especially omega-3, omega-6, and conjugated linoleic acid (CLA)—are recognized for their health advantages, which encompass the prevention of arteriosclerosis, coronary heart disease, and inflammatory disorders, in addition to their function in tumor development inhibition. Moreover, omega-3 fatty acids, especially docosahexaenoic acid (DHA), are crucial for the development of the child brain, eyes, and nervous system and promoting long-term cardiovascular health. These advantageous benefits have prompted investigations into alternate dietary approaches that might augment the UFA content in ruminant products without adversely affecting rumen fermentation and microbial communities [3,4].

Feeding oils to ruminants enhances energy intake, milk fat composition, and reproduction. Vegetable fats provide essential fatty acids, improving animal fertility and boosting productivity [5]. However, poly-unsaturated fatty acid-rich diets can affect nutrient digestibility and change rumen fermentation patterns, which might lead to less volatile fatty acid (VFA) generation and changes to microbial populations, especially biohydrogenation bacteria, like Butyrivibrio fibrisolvens and Butyrivibrio proteoclasticus, which may finally lead to a milk fat depression in ruminants [6,7]. Notably, fat in whole oilseeds is more ruminally inert than free oil due to slower fatty acid release [8]. This is why alternative forms of oil supplementation should be developed primarily to preserve the benefits of vegetable oils while mitigating the adverse effects of incorporating them as an ingredient in animal feed.

This unexplored but important nanotechnology application, oil-in-water nanoemulsions, may provide a potential solution to ruminant nutrition. Nanoemulsions are multiphase colloidal dispersions with droplet sizes below 100 nm, created by dispersing one immiscible liquid in another by physical shear-induced rupturing at the nanoscale [9]. Our team has been studying the potential of oil nanoemulsions in milk productivity and composition for some years. Notably, we observed a better unsaturated fatty acid profile (UFA) in the milk of ruminants that consume nanoemulsified edible oils, which helped skip biohydrogenation and increase the outflow of UFAs from the rumen. Because of their smaller droplet size, nanoemulsified oils are believed to have less effect on rumen fermentation and microbial populations than raw oils, leaving more UFA for absorption and incorporation into ruminant products [10,11,12,13]. However, the effect of nanoemulsified oil supplementation on in vivo rumen fermentation patterns, nutrient intake, and microbial population is not well-documented.

Given these considerations, the current research was conducted to assess the impact of raw (CO) and nanoemulsified (NCO) corn oil on lactating Barki ewes’ dry matter intake, nutrient digestibility, rumen fermentation, and rumen fatty acid composition during lactation. Results from this research may help understand the potential of this new form as an alternative to raw oil supplementation in ruminant nutrition.

## 2. Materials and Methods

In our previous research, which involved feeding nanoemulsified corn oil to lactating Zaraibi goats [12], we observed a significant impact on milk production and its fatty acid composition. However, due to inadequate management practices, we were unable to gather precise data on feed intake, rumen fermentation traits, and rumen fatty acid composition. Consequently, the current study was conducted in a managed farm of lactating Barki ewes, aiming to provide insights into nutrient intake, volatile fatty acids, and rumen fatty acid profiles, as well as a clearer understanding of the ruminal microbial populations involved in lipid metabolism within the rumen. Despite conducting the trial on a different species (due to availability), we ensured that all other aspects were replicated from our previous study.

### 2.1. Locations of the Study

The experiment with lactating Barki ewes was carried out in a private small-ruminant farm in Atfih, Giza, Egypt (29°25′0″ N, 31°15′0″ E). In the absence of established guidelines for the use and care of animals in scientific procedures in Egypt, the experimentation, handling, and management of sheep were conducted in accordance with the Polish Act for the Protection of Animals Used for Scientific and Educational Purposes (Dz. U. 26 January 2015, item 266).

### 2.2. Nanoemulsion Preparation

The corn oil–water emulsion was pre-mixed at 13,500 rpm using a digital high-speed homogenizer for 2 min (HG-15D Homogenizer, Daihan Scientific Co., Gangwon-Do, Republic of Korea) to achieve a droplet size distribution of 5.9 ± 0.4 µm. The corn oil-in-water nanoemulsion was prepared from the pre-homogenized solution using a Sonics VCX750 ultrasonic processor with a nominal power of 750 watts and a frequency of 20 kHz, equipped with a 25 mm sonotrode tip. The process was conducted at 80% amplitude for 20 min (Sonics and Materials, Newtown, CT, USA), resulting in a droplet size distribution ranging from 35 to 195 nm, with 90% of the distribution averaging 61 ± 9 nm. The technique described was executed in accordance with the findings of El-Sherbiny et al. [13]. The oil-in-water emulsion comprised 15% corn oil (sourced from the Egyptian market), 5.6% Tween 80 (Sigma Aldrich, Darmstadt, Germany), and 79.4% distilled water. A Zetasizer Nano ZS (Malvern Instruments, Malvern, UK) was employed to monitor the size of the generated nanoemulsion liquid suspension, assessed weekly at 25 °C.

### 2.3. Animal Management

Twenty-one multiparous lactating Barki ewes (mean ± SD: 3 ± 0.4 parity, 44.3 ± 1.9 kg body weight, 30 ± 2.7 months of age, and 402 ± 23 g/d of prior milk production) were randomly allocated to three dietary groups (*n* = 7 ewes/group) two weeks before the expected parturition in a completely randomized design. A power analysis was conducted based on milk production data, indicating that the total sample size of ewes ranged from 21 to 42, with associated power values spanning from 0.60 to 0.95. The implemented sample size was 21, which yielded a power value of 0.60, i.e., below the threshold typically recommended by statisticians. However, utilizing the resource equation approach, this sample size remains within an acceptable range of degrees of freedom for the analysis of variance (ANOVA), specifically between 10 and 20, which is deemed sufficient for drawing valid conclusions. Our primary objective was to replicate findings from our previous study on lactating goats, particularly focusing on intake and rumen fermentation patterns, which were not addressed in our last investigation. In the study by El-Sherbiny et al. [12], the sample size for goats varied from 21 to 39, with power values ranging from 0.70 to 0.95. The experiment was conducted with a sample size of 24, achieving a power of 0.80. Although we initially aimed to reach a power level of 0.80 in the current experiment, corresponding to a sample size of 27, the availability of ewes with similar productive and age characteristics was insufficient to sustain that power level and increase the group size. The experiment spanned 30 days, comprising a 25-day adaptation phase followed by a 5-day sampling phase. The sheep in each group were housed with their offspring in separate stalls (3 × 7 m each) and were fed according to treatment groups based on body weight as per the NRC [14]; however, to ensure the collection of orts, feed was provided at a rate 1.10 times greater than the NRC recommendation.

### 2.4. Diet and Treatments

The provided feed consisted of a 50% concentrate feed combination (pellets) and 50% roughage (berseem clover), as illustrated in Table 1. Subsequently, one week post-parturition, the lactating ewes were randomly assigned (7 ewes per group) to one of the following treatments: The Control group consisted of a baseline meal devoid of any supplementation, with a 50:50 ratio of concentrate to roughage, as detailed in Table 1. CO—the Control group diet supplemented with 3% corn oil in its raw form, computed on a dry matter basis; NCO—the Control group diet supplemented with 3% corn oil in nanoemulsified form, also measured on a dry matter basis. Lactating ewes were provided feed bi-daily at 7 a.m. and 7 p.m. The feed utilized for all treatments was evaluated in triplicate every ten days to assess the entire chemical composition, particularly dry matter, and to make adjustments as needed. The raw corn oil supplementation (CO) was combined with 150 g of concentrate and administered separately to each animal twice daily, before the morning and evening feedings, to guarantee that each animal received the designated quantity of oil supplementation. The nanoemulsified corn oil (NCO), manufactured daily, was split into four equal amounts and administered to the ewes using a 50 mL plastic syringe at three-hour intervals from 7 a.m. to 7 p.m.

The amount of CO administered was around 36 mL/ewe; however, when turned into nanoemulsion (which contains 15% of oils), this results in a total volume of around 240 mL/ewe of NCO. The NCO is mostly water (over 79%) which, if mixed with concentrate, can lose its nanoemulsion structure and benefits. That is why, in order to keep the nano-droplet size intact, it was decided that the NCO would be administrated oraly to the ewes, preferably in smaller portions throughout the day, to simulate our previous aim in using the nanoemulisifed oils in ruminant nutrition via their drinking water containers (nanoemulsions mixed with water). Samples of feed, rumen fluid, and feces were obtained during the sampling period. Table 2 displays the fatty acid content of the provided feed and supplements.

### 2.5. Feed Intake and Nutrient Apparent Digestibility

Three digestibility assays were performed over the final five days, utilizing acid-insoluble ash as an internal measure of indigestibility. The equations proposed by Ferret et al. [15] were employed to compute the coefficients of apparent digestion. Feed intake was determined by subtracting the orts from the feed provided on the preceding day. Fecal grab samples were obtained from each individual twice daily at 07:00 and 15:00 h, dried at 60 °C in a forced-air oven for 48 h, and pooled per ewe. Composite samples of desiccated feeds, orts, and feces were pulverized to pass through a 1-mm sieve using a mill and tested for dry matter, ash, nitrogen, and ether extract (EE) in accordance with AOAC [16] standard techniques. The NDF content was assessed following the methodology established by Van Soest et al. [17]. ADF content was assessed in accordance with AOAC [16] and expressed as exclusive residual ash, while the organic matter concentrations were computed.

### 2.6. Sampling and Analysis of Rumen Fluid

During days 26–30 of the experiment, ruminal fluid samples were obtained from all animals in the morning at 3 h post-feeding (at 10:00 h) to assess the amounts of volatile fatty acids and ammonia nitrogen. Approximately 100 mL of ruminal fluid was extracted from each ewe using a stomach tube, with the initial 50 mL of the samples removed to prevent saliva contamination, and the rumen contents were filtered through four layers of cheesecloth. The pH was promptly assessed utilizing a benchtop pH meter (Orion Star pH Meter, Thermo Fisher Scientific Inc., Rheinfelden, Germany). The ammonia nitrogen (NH3-N) concentration was quantified utilizing the colorimetric Nessler method, as reported in [11].

The volatile fatty acids (VFAs) were analyzed according to El-Sherbiny et al. [13] with minor modifications. Briefly, 0.8 mL of fermentation fluid and 0.2 mL of a solution containing 250 g of metaphosphoric acid/L were combined. Using a gas chromatograph (GC) with an automated sampler (Model 7890B; Agilent Technologies, Palo Alto, CA, USA), the concentration of VFA and its individual molar proportions were determined at the Central Laboratories Network, National Research Centre, Cairo, Egypt. The GC–MS was outfitted with a capillary column HP–FFAPv (19091F-112; 0.320 mm outer diameter, 0.50 m inner diameter, and 25 m length; J&W Agilent Technologies Inc., Palo Alto, CA, USA). As an external standard (Sigma Chemie GmbH, Stein, Germany), a mixture of known concentrations of individual short-chain fatty acids (acetate, propionate, and butyrate) was used to calibrate the integrator. The VFA peaks were identified qualitatively and quantitatively using external standards prepared by mixing Fluka-purchased individual VFA (Sigma Aldrich, St. Louis, MO, USA). MS Workstation 5.0 was utilized for data processing. The fatty acid methyl ester (FAME) analysis procedure was outlined in El-Sherbiny et al. [13] for the samples of fermentation fluid and dry ground feed; 2500 µL and 100 mg of fermentation fluid and dry feed samples, respectively, were added to 3 mL of 2 M NaOH for hydrolysis of the samples in a closed system employing 15 mL screw-cap Pyrex tubes with Teflon stoppers. The hydrolyzed samples were incubated in a block heater for 40 min at 90 °C. The extracted samples were then esterified in methanol with 0.5 M NaOH and converted to FAME in boron trifluoride (1.3 M; Fluka-Sigma Aldrich, St. Louis, MO, USA). A gas GC–MS system (7890B, Agilent, Santa Clara, CA, USA) was utilized, with a mass spectrometer detector and a 100 m fused silica capillary column (0.25 mm i.d., coated with 0.25 m Agilent HP; Chrompack CP7420; Agilent Technologies, Santa Clara, CA, USA) (5977A). Throughout the FAME chromatographic analysis, the carrier gas was hydrogen, which flowed at 1.3 mL/min. The injector and detector temperatures were 200 °C and 250 °C, respectively. The oven temperature was set to begin at 120 °C for 7 min, then increased by 7 °C per minute to reach 140 °C, where it remained for 10 min. It was later increased by 4 °C per minute to 240 °C, then 1 µL of the sample was injected into the GC Column. The peaks were identified by comparing their retention times to those of the relevant FAME standards (37 FAME Mix, Sigma Aldrich, West Chester, PA, USA) utilizing Open Lab CDS version 2.6. (Agilent, Santa Clara, CA, USA). In addition, the retention times of a reference standard and conjugated linoleic acid peaks were compared to identify them (a mixture of cis- and trans-9,11 and 10,12 octadecadienoic acid methyl esters; Sigma Aldrich, PA, USA), and the compositions of FA were given as g/100 g of total FA. The Central Service Unit at the National Research Centre in Egypt performed the chromatographic FA analyses.

Using real-time PCR, eight rumen bacterial species were quantified. According to El-Sherbiny et al. [13], metagenomic DNA was extracted from rumen fluid using a QIAamp DNA Stool mini kit (Qiagen GmbH, Hilden, Germany). Using the BLAST program 2.12.0 in the GenBank database, the specificity of primers was verified (Table 3). The QuantStudio 12 Flex PCR system quantified specific bacteria with a known initial DNA concentration (25 ng/L) (Life Technologies, Thermo Fisher Scientific, Waltham, MA, USA). The Power SYBR Green PCR Master Mix was utilized for PCR amplification (Thermo Fisher Scientific, Waltham, MA, USA). The reaction mixture contained 4 L of the 2X master mix, 25 ng of template DNA, and 0.5 M of each primer in a final volume of 10 L. The following amplification protocol was utilized: one cycle of amplification at 95 °C for 10 min for initial denaturation, 45 cycles at 95 °C for 15 s, followed by annealing at temperatures (dependent on the analyzed bacteria) for 5 s, and finally at 62 °C for 67 s. In the final phase of each cycle, fluorescent by-products were discovered. An analysis of product melting after a single amplification (0.1 °C increments from 65 °C to 95 °C with fluorescence collection at 0.1 °C intervals) was performed to explain the characteristics of amplification. Using the formula 2^−△△CT^ (RTA), the relative abundances of DNA copies of each bacterial species relative to the total bacteria were calculated.

### 2.7. Statistical Analysis

The PROC MIXED procedure of SAS (SAS^®^ OnDemand for Academics (Web-based SAS studio), 2024 SAS Institute Inc., Cary, NC, USA) was used to analyze all parameter data (VFA profile, FA profile, and microbial abundance) using a completely randomized design with a repeated measurements model as follows:

$$Y_{\mathrm{ijkl}}=\mu +T_{i}+A_{j}\;(T_{i})+D_{k}+{\left(T\;\times \;D\right)}_{\mathrm{ik}}+e_{\mathrm{ijkl}}$$

where Y_ijkl_ included each observation of the j ewe in k sampling time given i diet, T_i_ expressed the effect of diets, A_j_ (T_i_) expressed the ewe within each diet, D_k_ expressed the sampling day effect, (T × P)_ik_ expressed the interaction between the diets and sampling day, and e_ijkl_ expressed the experimental error. The day effect and diet × day interactions were nonsignificant (*p* > 0.05) for all measurements; thus, the statistical analysis was repeated using only the main effects of the diets. Multiple comparison Tukey tests were used to evaluate differences among the treatments. At *p* ≤ 0.05 and 0.05 < *p* ≤ 0.10, respectively, treatment effects were deemed significant or trending towards significance. The means and pooled standard errors of the means are displayed for all values.

## 3. Results

The results concerning milk productivity, composition, and fatty acid profiles followed the same pattern as those obtained in our previous study [12]. Briefly, the milk production and fat percentage were affected by CO feeding, which was significantly lower than NCO. Thus, we have opted not to present these results and, instead, to focus on the main goal: dry matter intake, nutrient digestibility, and rumen parameters (fermentation pattern, volatile fatty acid/fatty acid profile, and selected microbial abundances) to avoid repetition and confusion for the reader.

### 3.1. Dry Matter Intake and Nutrient Digestibility

The effects of using raw (CO) and nanoemulsified (NCO) corn oil in the nutrition of Barki ewes on dry matter intake and nutrient digestibility are presented in Table 4. The results indicated a decrease in dry matter intake (DMI) with CO; this reduction in DMI was associated with lower digestibilities of organic matter (OM), crude protein (CP), ether extract (EE), neutral detergent fiber (NDF), and acid detergent fiber (ADF) compared to the Control and NCO. Conversely, the NCO exhibited similar impacts on DMI as well as on the digestibilities of OM, CP, EE, NDF, and ADF when compared to the Control.

### 3.2. Rumen Basic Parameters and Volatile Fatty Acids

The effects of using raw corn oil (CO) and nanoemulsified corn oil (NCO) in the nutrition of Barki ewes on rumen fermentation parameters and volatile fatty acid profiles are presented in Table 4. The results indicated a significant reductive impact of CO on pH levels, ammonia-N, and total volatile fatty acids compared to the Control and NCO. CO also reduced acetate by 7.1% and 7.8%, butyrate by 11.2% and 14%, and propionate by 5.6% and 7.1% compared to the Control and NCO, respectively. In contrast, NCO did not alter the pH value, ammonia-N, or total volatile fatty acid concentrations; in addition, it did not affect the profiles of acetate, butyrate, and propionate compared to the Control.

### 3.3. Rumen Fatty Acid Profile

The rumen fatty acid composition of lactating Barki ewes fed raw corn oil (CO) and nanoemulsified corn oil (NCO) is presented in Table 5. It is evident that feeding NCO resulted in a lower profile of biohydrogenation-related fatty acids, particularly C18:0, C18:1 trans-10, and C18:1 trans-11, compared to the Control and CO. A decrease in conjugated linoleic acid isomers (CLA)- C18:2 cis-9 trans-11 and C18:2 trans-10 cis-12 was also observed with NCO when compared to the other treatments.

The total sum of saturated fatty acids (SFA) decreased significantly with NCO feeding compared to the Control and CO by 7.5% and 11.6%, respectively; NCO also yielded a higher sum of mono-unsaturated fatty acids (MUFA) by 18.8% and 33.5%, and a higher sum of poly-unsaturated fatty acids (PUFA) by 13.8% and 10.9% compared to Control and CO, respectively. Conversely, CO showed a significant increase in biohydrogenation-related fatty acids, especially the CLA isomers C18:2 cis-9 trans-11 and C18:2 trans-10 cis-12; however, higher SFA levels were also linked to CO compared to the Control and NCO.

### 3.4. Ruminal Microbial Population

Table 6 illustrates the results from the quantitative PCR analysis of specific ruminal microbial populations influenced by CO and NCO. Introducing NCO to the diet of lactating Barki ewes led to a notable increase (*p* < 0.05) in the relative abundance of Butyrivibrio fibrisolvens, Butyrivibrio proteoclasticus, and Ruminococcus albus when compared to both the Control and CO groups.

Additionally, NCO notably impacted (*p* < 0.05) the relative proportions of Anaerovibrio lipolytica and Streptococcus bovis. Nevertheless, Fibrobacter succinogenes just showed a tendency to increase with NCO when compared to the Control and CO groups.

## 4. Discussion

### 4.1. Dry Matter Intake and Nutrient Digestibility

The NRC [14] recommends that the dietary fat content in the diet of lactating ruminants should not surpass 6–7% of the provided dietary dry matter (DM). In our study, the overall fat level in the Control diet constituted approximately 4% of the average dry matter. Corn oil supplementation was determined to account for 3% of the dry matter; therefore, the fat content in the overall diet meets the required levels established by the National Research Council—USA. Nonetheless, the incorporation of raw corn oil appears to affect dry matter intake and nutrient digestibility significantly. It is well documented that including unsaturated fatty acid (UFA) rich oil in ruminant nutrition is associated with adverse effects on dry matter intake, nutrient digestibility, and, above all, a toxic load on rumen microbes [5,6,25]. It was reported that including vegetable oils high in linoleic acid (LA) in lambs’ diets drastically reduced the voluntary intake of dry matter and the digestibility of nutrients, specifically fiber [6,26]. Lima et al. [6] reported that the intake of dry matter, crude protein, NDF, and total digestible nutrients was reduced linearly with increasing levels of soybean oil inclusion in the lambs’ diet from 3% to 12% (on a DM basis). This matches the response obtained from feeding lactating Barki ewes 3% corn oil on a dry matter (DM) basis in our study. This results from high-UFA supplements, which typically increase the energy density in the feed, prompting the animal’s natural behavior to cease feeding once it has consumed enough energy [6]. It was also reported that diets high in UFA have the potential to stimulate the synthesis of cholecystokinin in the small intestine. This enzymatic hormone promotes rumen distension and satiety, which ultimately results in a reduction in feed intake. It does this by increasing the retention time in the rumen and reducing the time it takes to empty [6]. The decrease in nutrient digestibility has also been recorded with oil supplementation, which has the drawback of slowing feed fermentation in the rumen due to the oil coating the feed particles. Consequently, enzymes generated by bacteria were challenging in terms of infiltration of the feed particles [8]. The oil also possesses antibacterial effects. It may interfere with the rumen metabolic process, reducing feed digestibility [8,27]. On the other hand, protected oil was reported to avoid the adverse effects of unprotected oil supplementation on dry matter intake and nutrient digestibility [28]. This suggests that the similar effect obtained from feeding nanoemulsified corn oil (NCO) on the DM intake and nutrient digestibility compared to the Control in the current study reinforces our hypothesis that the nanoemulsified form of supplemented oils may have a protective potential, as supported by our previous findings [10,11,12,13]. It is worth noting that the high energy intake of NCO, which resulted from the greater dry matter intake and higher nutrient digestibility, demonstrated higher milk productivity compared to CO, which had lower energy intake.

### 4.2. Rumen Basic Parameters and Volatile Fatty Acids

Ruminal pH was influenced by raw corn oil supplementation, in line with Golbotteh et al. [29], who found that the pH was lower in cows fed 2% soybean oil than in those fed 2% fish oil. The same observation was also applicable in the case of ammonia-N, which was influenced more by soybean oil than the supplementation of fish oil [29]. However, it was clear that the NCO maintained a moderate pH and ammonia-N concentration compared to the Control. Ca-salt [30] was reported to have higher pH, acetate, propionate, and butyrate than corn oil, which may support our finding regarding NCO, which may have protected corn oil and reversed its negative impact on rumen fermentation and volatile fatty acid production. The study conducted by Martin et al. [31] has also highlighted that lower volatile fatty acid concentration, mainly acetate and propionate, with corn oil supplementation, aligns with lower fiber digestibility for this diet, which was also reported in our study.

### 4.3. Rumen Fatty Acid Profile and Microbial Populations

Khiaosa-ard et al. [32] used lipid emulsification to better distribute the fatty acids in rumen biohydrogenation trials. Their results showed that a three-minute ultrasonic bath dispersion of linoleic acid significantly changed the quantity of fatty acids accumulated post-incubation. They reasoned that fermentation fluid lipolysis and biohydrogenation would be less common in stable emulsions created by sonication because the small fatty acid droplets tended to stay in the liquid phase instead of binding to feed particles. However, our goals were different; initially, we wanted to ensure that nanoemulsions were more likely to be used as a supplement containing oils rich in poly-unsaturated fatty acids, ready to add to the drinking water for ruminant nutrition. It appears that NCO can prevent the biohydrogenation of a greater amount of UFA in the rumen (Table 5). The reduced fraction of biohydrogenation intermediates and the noticeably larger proportion of linoleic acid relative to CO and the Control made this point very evident. The nanoemulsion technology converts oils into a form that is less harmful to rumen bacteria, which is a good way to characterize the overall impact of NCO [10,11,12,13]. Possible explanations for the observed difference in activity between the nanoemulsified form of corn oil and the same amount of raw corn oil include the potential inhibition or bypass of ruminal lipolysis and ruminal biohydrogenation (as shown by the results of [18]) and the uptake of the rumen microbes to the small droplet of supplemented nanoemulsified soybean oils (as proposed by Bauchart et al. [33]). Nanoemulsions were irreplaceable in delivering oil-soluble vitamins, significantly increasing their bioaccessibility [34]. An increased level of unsaturation in fatty acids results in a more significant inhibitory effect on rumen bacterial populations. This is attributed to the ability of unsaturated fatty acids to embed themselves in the lipid bilayer of microbial cell membranes, causing disruption. For instance, certain rumen bacteria, including cellulolytic bacteria and some butyrate-producing species, show a reduction in numbers when exposed to any poly-unsaturated fatty acids (PUFAs) at a concentration of 50 µg/mL [35]. According to several studies [5,13,26], Ruminococcus albus and Ruminococcus flavefaciens are fibrolytic microbial bacteria heavily affected by oil supplementation; additionally, oils rich in UFA may decrease the abundance of the genus Butyrivibrio and Anaerovibrio lipolytica. In our study, corn oil supplementation followed the same pattern, decreasing the abundance of most of the detected rumen bacteria. On the other hand, research also indicated that protected fat is linked to a notable increase in the abundance of rumen bacteria. The study by Behan et al. [36] showed that sheep fed a diet supplemented with calcium soap of palm fatty acids exhibited a marked rise in F. succinogens and R. flavefaciens populations. This increase stands in contrast to those fed unprotected palm fatty acids or a standard Control diet, highlighting the significant impact of protected fats on rumen health and functionality. This finding aligns with those resulting from NCO in our study, in which nanoemulsions seem to preserve UFA without affecting the rumen microbial population.

## 5. Conclusions

The nanoscale droplets of corn oil, rich in poly-unsaturated fatty acids, have shown great potential in preserving higher levels of these beneficial fatty acids. Our data indicated that adding nanoemulsified forms of edible oil positively influences the levels of accumulated unsaturated fatty acids in the rumen, particularly n-3 and n-6 fatty acids. Significantly, feeding corn oil nanoemulsion does not adversely affect dry matter intake, nutrient digestibility, or the rumen fermentation process. Based on these findings, adding unsaturated lipids through nanoscale droplets of oil-in-water nanoemulsion could be a viable and efficient technique to supply oils to ruminant nutrition without negatively affecting the rumen fermentation capacity and microbial population composition.

## Figures and Tables

**Table 1:** Ingredients and chemical composition of the Control diet.

Item	Control Diet
Ingredients, g/kg of DM	
Corn grain	75.5
Cottonseed meal	116
Sunflower seed meal	85.5
Wheat bran	175
Molasse	35.5
Mineral-vitamin mixture ^1^	12.5
Berseem clover	500
Chemical composition, g/kg of DM	
Organic matter	909
Ash	91
Crude protein	160
Either extract	40
Neutral detergent fibre	372
Acid detergent fibre	213

^1^ Composition per 100 g: Vit. A (IU) 10,823, Vit. D3 (IU) 3322, Vit. E (mg) 35.25, Vit. B1 (mg) 4.4, Vit. B2 (mg) 1.25, Vit. B6 (mg) 3.95, Vit. B12 (mg) 26.1, Vit. C (mg) 4.05, Vit. K3 (mg) 3.9, Calcium pantothenate (mg) 5.2, Folic acid (mg) 2.95, Choline chloride (mg) 6.85, Biotin (mg) 4.65, Magnesium sulfate (mg) 399.2, Selenium (mg) 3.45, Zinc sulfate (mg) 454.6, Copper sulfate (mg) 329.4, Cobalt sulfate (mg) 4.2, Manganese (mg) 407.7, Iron sulfate (mg) 599.2, Iodine (mg) 3.55, Sodium chloride (g) 9.15.

**Table 2:** Fatty acid composition (g/100 g of FA) of the Control diet and supplements.

Item	Control	Supplements ^1^	
		CO	NCO
C14:0	0.23	0.14	0.19
C16:0	18.3	11.8	10.1
C18:0	2.67	2.81	2.49
C18:1 cis-9	28.3	27.1	28.9
C18:2 cis-9,cis-12	41.2	53.9	54.6
C18:3 cis-9,cis-12,cis-15	6.19	1.18	1.16
Other FA ^2^	3.11	3.07	2.56
SFA ^3^	23.5	15.5	13.4
UFA ^4^	76.5	84.5	86.6
MUFA ^5^	29.1	28.3	29.9
PUFA ^6^	47.4	56.2	56.7

^1^ Supplements: CO-corn oil, NCO-nanoemulsified corn oil; ^2^ Sum of other fatty acids including C6:0, C8:0, C10:1, C11:0, C12:0, C13:0, C14:1, C16:1, C19:0, C18:2 cis-9,cis-15, C21:0, C20:2, and C22:0; ^3^ Sum of saturated fatty acids; ^4^ Sum of unsaturated fatty acids; ^5^ Sum of mono-unsaturated fatty acids; ^6^ Sum of PUFAs.

**Table 3:** Forward (F) and reverse (R) primers used in the RT–PCR analysis of rumen bacteria.

Targeted Rumen Bacteria	Primer Sequence (5′ to 3′)	Reference
*Anaerovibrio lipolytica*	F: GAAATGGATTCTAGTGGCAAACG R: ACATCGGTCATGCGACCAA	[18]
*Butyrivibrio fibrisolvens*	F: ACACACCGCCCGTCACA R: TCCTTACGGTTGGGTCACAGA	[19]
*Butyrivibrio proteoclasticus*	F: TCCTAGTGTAGCGGTGAAATG R: TTAGCGACGGCACTGAATGCCTA	[20]
*Fibrobacter succinogenes*	F: GTTCGGAATTACTGGGCGTAAA R: CGCCTGCCCCTGAACTATC	[21]
*Megasphaera elsdenii*	F: AGATGGGGACAACAGCTGGA R: CGAAAGCTCCGAAGAGCCT	[22]
*Ruminococcus albus*	F: CCCTAAAAGCAGTCTTAGTTCG R CCTCCTTGCGGTTAGAACA	[23]
*Ruminococcus flavefaciens*	F: CGAACGGAGATAATTTGAGTTTACTTAGG R: CGGTCTCTGTATGTTATGAGGTATTACC	[24]
*Streptococcus bovis*	F: TTCCTAGAGATAGGAAGTTTCTTCGG R: ATGATGGCAACTAACAATAGGGGT	[22]

**Table 4:** Effect of the supplementation of raw and nanoemulsified corn oil on dry matter intake, nutrient digestibility, rumen basic parameters, and volatile fatty acid composition in Barki ewes.

Item	Control	CO ^1^	NCO ^2^	SEM	*p*-Value
*Dry matter intake and nutrient digestibility*					
Dry matter intake (DMI), kg/day	1.21 ^a^	1.11 ^b^	1.19 ^a^	0.009	0.002
Digestibility, g absorbed/kg ingested.					
Organic matter	631 ^a^	603 ^b^	639 ^a^	3.521	<0.001
Crude Protein	688 ^a^	649 ^b^	681 ^a^	3.872	0.005
Ether Extract	574 ^a^	541 ^b^	579 ^a^	3.845	<0.001
Neutral Detergent Fiber	601 ^a^	576 ^b^	598 ^a^	2.542	<0.001
Acid Detergent Fiber	539 ^a^	511 ^b^	529 ^a^	2.642	<0.001
*Rumen basic parameters and volatile fatty acid*					
pH	6.47 ^a^	6.39 ^b^	6.51 ^a^	0.011	<0.001
Ammonia-N, mmol/L	8.31 ^a^	8.11 ^b^	8.26 ^a^	0.019	<0.001
Total VFA, mmol/L	107 ^a^	98.4 ^b^	108 ^a^	0.990	0.003
Acetate (A), mmol/L	64.7 ^a^	60.1 ^b^	65.2 ^a^	0.523	0.005
Butyrate, mmol/L	24.1 ^a^	21.4 ^b^	24.9 ^a^	0.341	0.005
Propionate (P), mmol/L	17.9 ^a^	16.9 ^b^	18.2 ^a^	0.127	0.008
A:P ratio	2.68 ^b^	2.81 ^a^	2.62 ^b^	0.018	0.012

^a–b^ Means within a row with different superscripts differ (*p* < 0.05); ^1^ CO: Control diet + 3% corn oil in raw form; ^2^ NCO: Control diet + 3% corn oil in nanoemulsified form.

**Table 5:** Effect of the supplementation of raw and nanoemulsified corn oil on rumen fatty acid composition in Barki ewes.

Item	Control	CO ^1^	NCO ^2^	SEM	*p*-Value
C14:0	1.88 ^b^	1.97 ^a^	1.90 ^b^	0.011	<0.001
C14:1 cis-9	1.27 ^a^	1.15 ^b^	1.29 ^a^	0.017	<0.001
C16:0	16.9 ^a^	16.1 ^a^	14.3 ^b^	0.279	0.009
C16:1 cis-9	1.00 ^a^	0.90 ^b^	0.91 ^b^	0.012	0.009
C18:0	41.3 ^b^	48.2 ^a^	39.9 ^c^	0.950	<0.001
C18:1 trans-10	2.41 ^b^	2.89 ^a^	2.17 ^c^	0.079	0.008
C18:1 trans-11	2.08 ^a^	2.10 ^a^	1.89 ^b^	0.024	0.011
C18:1 cis-9	7.50 ^c^	8.13 ^b^	9.12 ^a^	0.174	0.002
C18:2 cis-9 cis-12	3.05 ^c^	3.12 ^b^	4.98 ^a^	0.234	<0.001
C18:2 cis-9 trans-11	0.67 ^b^	0.99 ^a^	0.58 ^c^	0.009	<0.001
C18:2 trans-10 cis-12	0.33 ^b^	0.40 ^a^	0.22 ^c^	0.002	<0.001
C18:3 cis-9 cis-12 cis-15	0.80 ^b^	0.72 ^c^	0.99 ^a^	0.029	<0.001
Other FA ^3^	20.8 ^a^	13.6 ^c^	19.4 ^b^	0.811	<0.001
SFA ^4^	73.8 ^b^	77.3 ^a^	68.3 ^c^	0.970	<0.001
UFA ^5^	26.2 ^b^	22.7 ^c^	31.7 ^a^	0.951	<0.001
MUFA ^6^	19.9 ^b^	16.3 ^c^	24.5 ^a^	0.879	0.002
PUFA ^7^	6.23 ^c^	6.44 ^b^	7.23 ^a^	0.113	0.002

^a–c^ Means within a row with different superscripts differ (*p* < 0.05); ^1^ CO: Control diet + 3% corn oil in raw form; ^2^ NCO: Control diet + 3% corn oil in nanoemulsified form; ^3^ Sum of other fatty acid including C6, C10:1, C11:0, C16:1 trans, C18:1 trans-5, C18:1 trans-9, C18:1 cis-11, C18:1 cis-12, C18:1 cis-14, C18:1 cis-15, C18:2 cis-9 cis-15, C19:0, C20:1 trans, C21:0, C20:2, C22:0, C23:0, C22:2, C24:0, and C24:1; ^4^ Sum of saturated fatty acids; ^5^ Sum of unsaturated fatty acids; ^6^ Sum of mono-unsaturated fatty acids; ^7^ Sum of PUFAs.

**Table 6:** Effect of raw and nanoemulsified corn oil supplementation on ruminal microbial populations quantified using quantitative PCR.

Item	Control	CO ^1^	NCO ^2^	SEM	*p*-Value
*Anaerovibrio lipolytica*	1.24 ^a^	1.06 ^b^	1.31 ^a^	0.019	<0.001
*Butyrivibrio fibrisolvens*	1.44 ^a^	1.23 ^b^	1.46 ^a^	0.018	<0.001
*Butyrivibrio proteoclasticus*	2.98 ^a^	2.79 ^b^	3.11 ^a^	0.023	<0.001
*Fibrobacter succinogenes*	3.99	3.87	4.09	0.015	0.069
*Megasphaera elsdenii*	0.79 ^a^	0.63 ^b^	0.78 ^a^	0.013	0.002
*Ruminococcus flavefaciens*	1.01 ^a^	0.87 ^b^	0.99 ^a^	0.010	0.003
*Ruminococcus albus*	0.12 ^b^	0.07 ^c^	0.16 ^a^	0.003	0.002
*Streptococcus bovis*	0.06 ^a^	0.03 ^b^	0.06 ^a^	0.001	0.006

^a–c^ Means within a row with different superscripts differ (*p* < 0.05); ^1^ Rumen bacteria expressed as an arbitrary unit; ^1^ CO: Control diet + 3% corn oil in raw form; ^2^ NCO: Control diet + 3% corn oil in nanoemulsified form.

## Data Availability

The data presented in this study are available upon request from the corresponding author.
